# A nested case–control study of stomach cancer in relation to green tea consumption in Japan

**DOI:** 10.1038/sj.bjc.6601512

**Published:** 2004-01-06

**Authors:** Y Hoshiyama, T Kawaguchi, Y Miura, T Mizoue, N Tokui, H Yatsuya, K Sakata, T Kondo, S Kikuchi, H Toyoshima, N Hayakawa, A Tamakoshi, Y Ohno, T Yoshimura

**Affiliations:** 1 Department of Public Health, Showa University School of Medicine, 1-5-8 Hatanodai, Shinagawa, Tokyo 142-8555, Japan; 2 Department of Nursing, Saitama University, Saitama, Japan; 3 Department of Clinical Epidemiology, Institute of Industrial Ecological Sciences, University of Occupational and Environmental Health, Fukuoka, Japan; 4 Department of Public Health/Health Information Dynamics, Field of Social Life Science, Program in Health and Community Medicine, Nagoya University Graduate School of Medicine, Nagoya, Japan; 5 Department of Public Health, Wakayama Medical University, Wakayama, Japan; 6 Department of Public Health, Aichi Medical University, Aichi, Japan; 7 Department of Epidemiology, Research Institute for Radiation Biology and Medicine, Hiroshima University, Hiroshima, Japan; 8 Department of Preventive Medicine/Biostatistics and Medical Decision Making, Field of Social Life Science, Program in Health and Community Medicine, Nagoya University Graduate School of Medicine, Nagoya, Japan

**Keywords:** green tea, stomach cancer, JACC Study

## Abstract

To evaluate whether green tea consumption provides protection against stomach cancer, the relative risks (RRs) were calculated in the Japan Collaborative Study for Evaluation of Cancer Risk, sponsored by the Ministry of Health and Welfare (JACC Study). The study was based on 157 incident cases and 285 controls aged 40–79 years. Cox proportional hazards regression analysis was used to estimate the RRs for stomach cancer. It was found that green tea consumption had no protective effect against stomach cancer. After adjustment for age, smoking status, *H. pylori* infection, history of peptic ulcer, and family history of stomach cancer along with certain dietary elements, the risks associated with drinking one or two, three or four, five to nine, and 10 or more cups of green tea per day, relative to those of drinking less than one cup per day, were 1.3 (95% confidence interval (CI): 0.6–2.8), 1.0 (95% CI: 0.5–1.9), 0.8 (95% CI: 0.4–1.6), and 1.2 (95% CI: 0.6–2.5), respectively (*P* for trend=0.899). We found no inverse association between green tea consumption and the risk of stomach cancer.

Stomach cancer is the second most common cancer worldwide (Parkin *et al*, 1999). In Japan, this cancer is the leading cause of cancer death among women and the second among men (Statistics and Information Department, Minister's secretariat, Ministry of Health and Welfare Japan, 2000). It has recently been reported that green tea consumption is inversely associated with the risk of stomach cancer; in other words, it has a protective effect. Green tea polyphenols have various anticarcinogenic effects, such as strong antioxidant activity, and inhibition of nitrosation and cell proliferation.

Although case–control studies (Kono *et al*, 1988; Yu and Hsieh, 1991; Yu *et al*, 1995; Ji *et al*, 1996; Inoue *et al*, 1998; Setiawan *et al*, 2001) have found a reduced risk of stomach cancer in association with green tea consumption, prospective studies (Galanis *et al*, 1998; Nakachi *et al*, 2000; Nagao *et al*, 2001; Tsubono *et al*, 2001) have not. A recent prospective study found that green tea had a protective effect against stomach cancer. Urinary tea polyphenols have been associated with protection from the risk of stomach cancer, while controlling *Helicobacter pylori* infection. Past studies did not consider the presence or absence of a history of infection with *H. pylori*, a strong risk factor for stomach cancer (Asaka *et al*, 1997). Assuming that green tea consumption is related to *H. pylori* infection, when a subject has a history of infection with *H. pylori* and consumes a large quantity of green tea, the protective effect, if any, would be masked. The present nested case–control study aimed to examine the association between green tea consumption and the risk of stomach cancer, while controlling *H. pylori* infection and other potential confounders, using data from the Japan Collaborative Cohort (JACC) Study, a Japan-wide population-based prospective study. This is the first study to analyse the effects of green tea consumption while controlling *H. pylori* infection.

## MATERIAL AND METHODS

### JACC Study

This study was part of the Japan Collaborative Cohort Study for Evaluation of Cancer Risk sponsored by the Ministry of Education, Science, Sports and Culture of Japan (the JACC Study), a nation-wide multicentre collaborative study to prospectively evaluate various risk or protective factors for cancer mortality and incidence. Details of the study design were reported previously. Briefly, the cohort included 110 792 men and women (46 465 and 64 327, respectively), aged 40–79 years at recruitment, who were enrolled from 1988 to 1990. Enrollment was based on the participants of a general health checkup periodically provided by the 45 municipalities involved. The informed consent procedures were approved by the Ethics Committee of Medical Care and Research, University of Occupational and Environmental Health, Kitakyushu, and the Ethical Board of the Nagoya University School of Medicine, Japan.

At the time of recruitment, baseline characteristics were gathered by a self-administered questionnaire, which covered the medical history and included items such as drinking and smoking, level of education, and family history of several medical conditions including cancer. About one-third of the cohort members (*n*=39 293) also donated a residual serum sample (about 2 ml) to be used for the general health checkup. This sample was partitioned into 0.3–0.5 ml aliquots and stored at −80°C, until laboratory analyses were performed. The *H. pylori* antibody level was measured in the serum using HM-CAPTM (Enteric Products, Westbury, NY, USA) with an antigen from Japanese (J-HM-CAP). The cutoff value was determined at 2.3, which was recommended in the manufacturer's instructions.

### Follow-up and identification of stomach cancer cases, and selection of control subjects

The vital status of each participant was checked annually by each regional research centre, with permission from the Ministry of Public Management, Home Affairs, Post and Telecommunications to review their population register sheets. The incidence of cancer was ascertained in 24 study areas (*n*=65 184) and coded according to the tenth revision of the International Classification of Disease and the second edition of the International Classification of Diseases for Oncology. These data were collected at the central office of the Research Committee.

We first restricted the subjects to those who lived in the study areas where cancer incidence was ascertained. We then excluded 857 participants with a self-reported history of cancer. From the remaining 64 327 subjects, diagnosis of stomach cancer at 12 or more months after cohort recruitment was documented in 804 subjects until the end of 1997. Serum had been obtained from 218 cases of the initial 804 cases. However, seven cases without sufficient serum for the laboratory analyses and one case without an eligible control subject were excluded. Thus, the study reported here included 210 cases in total. There were no differences between those selected for the case–control study nested within the cohort and those who were not selected in terms of the variables included in the multivariate model. The lag time between blood sampling and stomach cancer diagnosis varied between 12 and 113 months (median, 50 months). Each of these subjects was matched with two control subjects with respect to sex, age at recruitment (as near as possible), and study area, who had also provided an adequate baseline blood sample, and who were alive and free of confirmed cancer by the end of 1997. Owing to a lack of eligible subjects, a few sets (*n*=10) contained only one control, and thus there was a total of 410 controls.

Since questions on the daily consumption of green tea were not included in the questionnaire in seven areas (four rural areas and three urban/rural areas), we excluded these data (49 cases and 88 controls). Of the 161 cases and 322 controls remaining, eight cases (5.0%) and 38 controls (11.8%) had green tea consumption data missing from the questionnaire; so these too were excluded. Owing to a lack of eligible subjects, 16 sets were further excluded. The remaining 151 cases and 265 controls were included in the present analysis.

### Data processing

Cox proportional hazard regression analysis was used. The relative risk (RR) and its 95% confidence interval (CI) were calculated based on the regression coefficient and its standard error (Cox, 1972), for an indicator term corresponding to the level of an independent variable. For multivariate analysis, several factors were listed as potential confounders according to epidemiological studies (Boeing, 1991; Hoshiyama and Sasaba, 1992; World Cancer Research Fund, 1997; Hoshiyama *et al*, 2002; Yatsuya *et al*, 2002). Trends of association were assessed by the regression model assigning scores (0–4) to the levels of the independent variables. Statistical significance (two-sided) was based on the ratio of the regression coefficient and its standard error. Statistical analysis (PHREG procedure) was performed using the Statistical Analysis System (SAS Institute, 1983).

## RESULTS

Table 1

**Table 1:** Characteristics of cases and controls

	**Case**	**Control**
**No**	**151**	**265**
Age (years)	61.7	61.5
*Green tea (cups per day)*		
<1	18	31
1 or 2	19	23
3 or 4	41	73
5–9	50	105
⩾10	23	33
*H. pyroli infection (%)*	88.7	77.7
History of peptic ulcer (%)	19.7	18.2
Family history of stomach cancer (%)^a^	20.5	16.2
⩽9 years of schooling (%)^b^	37.1	32.0
*Smoking (%)*		
Current	32.2	28.7
Past	18.5	18.0
*Daily dietary consumption (%)*		
Rice (⩾4 bowls day^−1^)	36.5	31.8
Miso soup (⩾1 cup day^−1^)	83.7	77.9
Preference for salty foods (yes)	33.8	29.6
Green-yellow vegetables (⩾1 day^−1^)	46.7	42.3
White vegetables (⩾1 day^−1^)	38.6	40.5
Fruits (⩾3 week^−1^)	44.4	39.3

a We defined a positive family history of stomach cancer as when the subject had a least one first-degree relative (parents or siblings) with a history of stomach cancer.

b Information on educational level was measured as the age of formal schooling completed and was classified into two categories: ⩽15 years old (corresponds to ⩽9 years of schooling) and ⩾16 years old (corresponds to ⩾10 years of schooling).

compares the characteristics of the cases and the controls. The consumption of green tea varied substantially. The proportion with a history of *H. pylori* infection was higher for the cases than for the controls. The proportion with a family history of stomach cancer was also higher for the cases than for the controls. The proportion of current smokers was also higher for the cases than for the controls. The cases consumed rice, miso soup, green–yellow vegetables, and fruit more frequently than the controls.

Table 2

**Table 2:** Relative risk of stomach cancer according to green tea consumption

	**Green tea consumption (cups day^−1^)**					
	**<1**	**1 or 2**	**3 or 4**	**5–9**	**⩾10**	***P* for trend**
Case/controls	18/31	19/23	41/73	50/105	23/33	
Age/sex-adjusted RR	1.0	1.2 (0.5–2.9)	0.9 (0.4–1.9)	0.7 (0.3–1.5)	1.0 (0.4–2.4)	0.515
Age/sex- and *H. pylori*-adjusted RR	1.0	1.1 (0.4–2.9)	0.9 (0.4–1.9)	0.7 (0.3–1.5)	1.1 (0.4–2.5)	0.628
Multivariate RR^a^	1.0	1.3 (0.6–2.8)	1.0 (0.5–1.9)	0.8 (0.4–1.6)	1.2 (0.6–2.5)	0.899

a Adjusted for age (four classes), smoking status (never, past, current), sex, *H. pylori* infection, history of peptic ulcer, family history of stomach cancer, educational level (two levels), consumption of rice, miso soup, green–yellow vegetables, white vegetables, fruits, and preference for salty foods (two categories). Values in parantheses are 95% confidence intervals. RR=relative risk.

shows the RR and its CI for stomach cancer according to green tea consumption. The age/sex-adjusted RRs associated with drinking one or two, three or four, five to nine, and 10 or more cups of green tea per day, relative to those of drinking less than one cup per day, were 1.2 (95% CI: 0.5–2.9), 0.9 (95% CI: 0.4–1.9), 0.7 (95% CI: 0.3–1.5), and 1.0 (95% CI: 0.4–2.4), respectively. Multivariate RRs were similar to age/sex-adjusted and age/sex- and *H. pylori* infection-adjusted RRs.

Table 3

**Table 3:** Relative risk of *H. pylori* infection positive according to green tea consumption among controls

	**Green tea (cups day^−1^)**					
	**<1**	**1 or 2**	**3 or 4**	**5–9**	**⩾10**	**P for trend**
Age–sex-adjusted RR of *H. pylori* infection positive	1.0	1.0 (0.2–3.8)	1.0 (0.3–2.8)	1.1 (0.4–3.1)	0.7 (0.2–2.5)	0.901

shows the age/sex-adjusted RRs of *H. pylori* infection positivity according to green tea consumption. *H. pylori* infection did not differ with the consumption of green tea.

## DISCUSSION

This nested case–control study is the first study to investigate any association between green tea consumption and the risk of stomach cancer while controlling *H. pylori* infection. Among the possible limitations of the present study was incomplete data. About 10% of subjects were excluded from the analysis because they had not provided information concerning their daily consumption of green tea. We could not fully evaluate the effects of the exclusion of these data. Nevertheless, there was no difference between the percentages of smokers in the whole data (53.1% of men and 2.9% of women) and those in the included data (51.9% and 3.7%, respectively), as examined by the Cochran–Mantel–Haenszel *χ*^2^ test (*P*=1.000 and 0.843, respectively). The missing information therefore seemed to occur randomly.

The second possible problem with the present study was in the questionnaire. The original words of the question on green tea were: Do you drink Japanese tea (green tea)? There are various kinds of Japanese tea, although for Japanese people green tea is the one that most often comes to mind. About 89% of the total production of Japanese tea in 1999 was ordinary green tea (The Ministry of Agriculture, Forestry, and Fisheries, 1999). We believe that a slight misclassification could have derived from the idiosyncrasy of our questionnaire pertaining to Japanese tea (green tea).

Green tea is widely consumed in Japan and other Asian countries. If drinking green tea protects against stomach cancer, it would be an inexpensive and convenient method of primary prevention. Tsubono *et al* reported that there was no association between green tea consumption and the risk of stomach cancer, consistent with the finding of this prospective study. Little other evidence is available from prospective studies (Galanis *et al*, 1998; Tsubono *et al*, 2001). Past studies did not consider the influence of *H. pylori* infection. Subjects with chronic gastritis caused by *H. pylori* infection might have limited their consumption of green tea. If so, the prevalence of infection would have been lower in the subjects with higher intakes of green tea. If not, the prevalence of infection would have been higher among the subjects with higher intakes of green tea. This condition would have masked an inverse association between the risk of stomach cancer and green tea consumption. We examined the association of *H. pylori* infection and green tea consumption, and found that *H. pylori* infection did not differ with the consumption of green tea (see Table 3).

Our findings are in general agreement with those of four prospective studies which found no inverse association between green tea consumption and the risk of stomach cancer (Galanis *et al*, 1998; Nakachi *et al*, 2000; Nagao *et al*, 2001; Tsubono *et al*, 2001). The number of cases of stomach cancer was relatively large in three studies. Recently, another prospective study was conducted in Shanghai, China. Urinary EGC positivity showed a statistically significant inverse association with stomach cancer (OR=0.52, 95% CI=0.28–0.97) after adjustment for *H. pylori* seropositivity, smoking, alcohol drinking, and the level of serum carotenes (Sun *et al*, 2002). Cumulative excretion of EGC increased with increasing green tea consumption in human subjects (Yang *et al*, 1998). It might be important to evaluate biomarkers of tea polyphenol exposure.

In summary, we found no inverse association between the consumption of green tea and the risk of stomach cancer in Japan in a nested case–control study.

## Appendix

### JAPAN COLLABORATIVE COHORT STUDY GROUP

The investigators involved in the present JACC study and their affiliations are as follows: Dr Yoshiyuki Ohno, (the present chairman of the Monbusho ECC), Dr Akiko Tamakoshi (Secretary General of the Monbusho ECC), and Dr Hideaki Toyoshima, Nagoya University Graduate School of Medicine; Dr Mitsuru Mori, Sapporo Medical University School of Medicine; Dr Yutaka Motohashi, Akita University School of Medicine; Dr Shigeru Hisamichi, Tohoku University Graduate School of Medicine; Dr Yosikazu Nakamura, Jichi Medical School; Dr Takashi Shimamoto, Institute of Community Medicine, University of Tsukuba; Dr Haruo Mikami, Chiba Cancer Center; Dr Shuji Hashimoto, School of Health Sciences and Nursing, University of Tokyo; Dr Yutaka Inaba, Juntendo University School of Medicine; Dr Heizo Tanaka, Medical Research Institute, Tokyo Medical and Dental University; Dr Yoshiharu Hoshiyama, Showa University School of Medicine; Dr Hiroshi Suzuki, Niigata University School of Medicine; Dr Hiroyuki Shimizu, Gifu University School of Medicine; Dr Shinkan Tokudome, Nagoya City University Medical School; Dr Yoshinori Ito, Fujita Health University School of Health Sciences; Dr Akio Koizumi, Graduate School of Medicine and Faculty of Medicine, Kyoto University; Dr Takashi Kawamura, Kyoto University Center for Student Health; Dr Yoshiyuki Watanabe, Kyoto Prefectural University of Medicine, Research Institute for Neurological Diseases Geriatrics; Dr Masahiro Nakao, Kyoto Prefectural University of Medicine; Dr Takaichiro Suzuki, Research Institute, Osaka Medical Center for Cancer and Cardiovascular Diseases; Dr Tsutomu Hashimoto, Wakayama Medical University; Dr Takayuki Nose, Tottori University Faculty of Medicine; Dr Norihiko Hayakawa, Research Institute for Radiation Biology and Medicine, Hiroshima University; Dr Takesumi Yoshimura, Institute of Industrial Ecological Sciences, University of Occupational and Environmental Health, Japan; Dr Katsuhiro Fukuda, Kurume University School of Medicine; Dr Tomoyuki Kitagawa, Cancer Institute of Japanese Foundation for Cancer Research; Dr Toshio Kuroki, Institute of Molecular Oncology, Showa University; Dr Naoyuki Okamoto, Kanagawa Cancer Center; Dr Teruo Ishibashi, Asama General Hospital; Dr Hideo Shio, Shiga Medical Center and Dr Kazuo Tajima, Aichi Cancer Center Research Institute.

The former investigators involved in the JACC study and their affiliations are as follows: Dr Kunio Aoki, Aichi Cancer Center; Dr Suketami Tominaga, Aichi Cancer Center Research Institute; Dr Sadamu Anzai, Dr Takeshi Kawaguchi, Dr Kenichi Nakamura, Dr Motofumi Masaki, Showa University School of Medicine; Dr Shuugo Kanamori, Dr Masachika Morimoto, Dr Seishi Yoshimura, Shiga Medical Center for Adults; Dr Sigetosi Kamiyama, Dr Yukio Takizawa, Dr Noriyuki Hachiya, Akita University School of Medicine; Dr Keiichi Kawai, Dr Shuichi Nakagawa, Dr Hiroki Watanabe, Kyoto Prefectural University of Medicine; Dr Minoru Kurihara, Research Institute for Radiation Biology and Medicine, Hiroshima University; Dr Yoshio Komachi, Institute of Community Medicine, University of Tsukuba; Dr Ruichiro Sasaki, Aichi Medical University; Dr Minoru Sugita, Toho University School of Medicine; Dr Iwao Sugimura, Asahikawa Kosei Hospital; Dr Toshihiko Tanaka, Chigasaki Public Health Center; Dr Tomio Hirohata, Kyushu University School of Medicine; Dr Isaburo Fujimoto, Center for Adult Diseases, Osaka; Dr Minoru Matsuzaki, Chigasaki Public Health and Welfare Center; Dr Hirotsugu Miyake, Sapporo Medical University School of Medicine; Dr Motoi Murata, Chiba Cancer Center; Dr Shinsuke Morio, Kanagawa Cancer Center; Dr Hiroshi Yanagawa, Jichi Medical School and, Dr Shaw Watanabe, Tokyo University of Agriculture.
